# The Impact of Cognition on Motor Learning and Skill Acquisition Using a Robot Intervention in Infants With Cerebral Palsy

**DOI:** 10.3389/frobt.2022.805258

**Published:** 2022-02-25

**Authors:** Raghuveer Chandrashekhar, Hongwu Wang, Josiah Rippetoe, Shirley A. James, Andrew H. Fagg, Thubi H. A. Kolobe

**Affiliations:** ^1^ Department of Occupational Therapy, College of Public Health and Health Professions, University of Florida, Gainesville, FL, United States; ^2^ Department of Rehabilitation Sciences, College of Allied Health, University of Oklahoma Health Sciences Center, Oklahoma City, OK, United States; ^3^ Department of Computer Science, University of Oklahoma, Norman, OK, United States; ^4^ Institute of Biomedical Engineering, Science, and Technology, University of Oklahoma, Norman, OK, United States

**Keywords:** cognition, robot, human robot interaction interface, motor learning, cerebral palsy

## Abstract

**Background:** Cerebral Palsy (CP) is a neurodevelopmental disorder that encompasses multiple neurological disorders that appear in infancy or early childhood and persist through the lifespan of the individual. Early interventions for infants with CP utilizing assisted-motion robotic devices have shown promising effects in rehabilitation of the motor function skills. The impact of cognitive function during motor learning and skill acquisition in infants using robotic technologies is unclear.

**Purpose:** To assess the impact of cognitive function of infants with and without CP on their motor learning using the Self-Initiated Prone Progression Crawler (SIPPC) robot.

**Methods:** Statistical analysis was conducted on the data obtained from a randomized control trial in which the movement learning strategies in infants with or at risk for CP was assessed during a 16-week SIPPC robot intervention. Cognitive function was measured by the Bayley scales of Infant and Toddler Development–Third edition (Bayley-III) and motor function was measured by the Movement Observation Coding Scheme (MOCS). The infants were categorized into three distinct groups based on their cognitive scores at baseline: “above average” (n_1_ = 11), “below average” (n_2_ = 10), and “average” (n_3_ = 26). Tri-weekly averages of the MOCS scores (observations at five time points) were used for the analyses. This study involved computing descriptive statistics, data visualization, repeated measures analysis of variances (rmANOVA), and survival analyses.

**Results:** The descriptive statistics were calculated for the MOCS and Bayley III scores. The repeated measures ANOVAs revealed that there was a statistically significant effect of time (*p* < 0.0001) on scores of all subscales of the MOCS. A statistically significant effect of interaction between group and time (*p* < 0.05) was found in MOCS scores of subscales 1 and 2. The survival analyses indicated that infants in different cognition groups significantly differed (*p* < 0.0001) in their ability to achieve the crawling milestone within the 16-week intervention period.

**Conclusion:** The findings in this study reveal the key movement strategies required to move the SIPPC robot, assessed by the MOCS, vary depending on the infants’ cognition. The SIPPC robot is well-matched to cognitive ability of infants with CP. However, lower cognitive ability was related to delayed improvement in their motor skills.

## Background and Significance

Cerebral Palsy (CP) is a neurodevelopmental disorder that encompasses multiple neurological disorders that appear in infancy or early childhood and persist through the lifespan of the individual (Peter, 2007). Population-based studies across the world report prevalence estimates of CP within the range of 1.5 to more than 4 per 1,000 live births (Arneson et al., 2009; Bhasin et al., 2006; CDCdataforCP, 2020; van Gorp et al., 2020; NINDSWebsite, 2019). The CDC’s Autism and Developmental Disabilities Monitoring Network reports one in 323 children in the United States have CP (CDCdataforCP, 2020). In 2000, estimates of lifetime cost encompassing all children born with CP in the United States was $11.5 billion (Honeycutt et al., 2004) and although the prevalence has not changed significantly in the past 10 years, the cost of care associated with CP has increased (Novak et al., 2013; Oskoui et al., 2013; van Gorp et al., 2020). CP continues to be the most physically disabling condition in the United States and among numerous other complications experienced by adults and children with CP, the most disabling is impaired mobility (Turk, 2009; McAdams and Juul, 2011). Children with CP experience some or all of the following symptoms: poor muscle coordination (ataxia), muscle spasticity, impaired postural control, upper or lower extremity weakness, tremors, delays in reaching motor skill milestones (independent crawling and walking), toe walking, crouched or adducted “scissored” gait, altered muscle tone, excessive drooling, and difficulty swallowing or speaking, and lack of manual dexterity (Oskoui et al., 2013; Turk, 2009; NINDSWebsite, 2019). These impairments impact these children’s motor, cognitive, psychological, and social development (Hadders-Algra, 2000; Anderson et al., 2013).

Because CP is caused by an irreversible injury to the brain, there are no currently known cures. Intervention strategies focus on maintaining and improving mobility, quality of life, function, and prevention of secondary complications. Cognitive impairments foster motor and functional impairments, which further exacerbate the cognitive impairment, resulting in a cycle of debilitating symptoms (Monteiro et al., 2010; Robert et al., 2013; Stadskleiv, 2020). Because fundamental functional ability and motor skills develop early within typically developing children, it is critical to initiate intervention early for children/infants at risk for, or diagnosed with CP (Shonkoff and Meisels, 2000; Bayon et al., 2016). Also essential, is incorporating quality of movement and functional activities within these intervention strategies, while encouraging interaction within a variety of environments to foster comprehensive development (Shonkoff and Meisels, 2000; Bayon et al., 2016; Stadskleiv, 2020). A variety of early intervention strategies are used to address complications associated with CP, and include physical therapy (PT), occupational therapy (OT), oral medication or botulinum toxin pumps or injections for spasticity, orthotics, and surgery. Depending on the type of CP, successful interventions can also include partial bodyweight supported treadmill training, constraint induced movement therapy, and robot assisted therapy (Novak et al., 2013; Robert et al., 2013; Bayon et al., 2016; Sadowska et al., 2020). While the outcomes are inconsistently reported (Novak et al., 2013), the literature does indicate intervention focusing on motor learning, partial bodyweight supported treadmill training, constraint induced movement therapy, and robot assisted therapy are more effective in enhancing functional mobility outcomes (Novak et al., 2013; Bayon et al., 2016; Kolobe and Fagg, 2019).

Among the early interventions involving motor learning, assisted-motion robotic devices such as the CPWalker, PALMIBER vehicle, and Self-Initiated Prone Progression Crawler (SIPPC), have demonstrated promise in rehabilitating functional mobility skills in infants with CP (Raya et al., 2015; Kolobe and Fagg, 2019). However, a common limitation of robotic devices is the complexity of the intervention devices, which makes them difficult to use when the children have cognitive impairments (Hogan and Krebs, 2004; Krebs and Hogan, 2006; Bayon et al., 2016). Since individuals with different levels of cognition learn the same task at varying paces, it is necessary to develop assistive devices that can be used by individuals with dissimilar levels of cognition. The CPWalker is a comprehensive robotic platform that consists of a smart walker with body weight and autonomous locomotion support, a wearable exoskeleton robot, and a motor neuroprosthesis for joint range of motion support, controlled by a multimodal human-robot interface (Raya et al., 2015). The multimodal human-robot interface used to control the CPWalker allows the integration of the peripheral nervous system (PNS) as well as the central nervous system (CNS), thus combining the physical (PNS) and cognitive (CNS) approach to motor rehabilitation in children with CP (Raya et al., 2015). The PALMIBER vehicle is a pre-industrial robotic vehicle designed and developed with an “assist as needed” paradigm and a playful interface. The PALMIBER vehicle promotes interaction between the child with CP and their environment through mobility experiences. Similar to the CPWalker, the PALMIBER vehicle also integrates a physical and cognitive approach in addressing motor impairments caused by CP (Raya et al., 2015).

The SIPPC robot differs from the CPWalker and the PALMIBER because it allows early intervention using robotics for infants with or *at risk* of developing CP (Ghazi et al., 2016; Kolobe and Fagg, 2019). Additionally, the SIPPC robot takes advantage of each infant’s self-initiated movement, critical for early locomotion and crucial for enhancing synaptic connection within the brain during the early stage of development (de Vries and de Groot, 2002; Kolobe and Fagg, 2019). The primary driving forces behind the conceptualization, design, and function of the SIPPC robot are two motor learning mechanisms available within the infant central nervous system: reinforcement learning (RL) and error-based learning (EBL). The SIPPC robot uses a physical and cognitive approach to rehabilitation, however it is uniquely designed to capture and enhance movement effort when infants are developing prone locomotion (crawling), one of the major mobility milestones in infant development (Miller et al., 2015; Raya et al., 2015; Kolobe and Fagg, 2019).

While robotic devices have shown promise in the rehabilitation of children with CP, the impact of cognitive function during motor learning and skill acquisition in infants using robotic technologies is still unclear. Understanding the impact of cognitive function on the outcomes of robotic intervention will guide the design and configuration of the human-robot interface. The aim of this study is to evaluate the effect of cognitive ability of infants with and without CP on their proficiency in learning SIPPC robot mobility (Miller et al., 2015; Ghazi et al., 2016; Kolobe and Fagg, 2019). In addition to informing future revision of the SIPPC robot, findings from this study will illustrate the interplay between robotic movement, learning, and cognition, as well as the levels of end-user cognitive ability necessary for the use of this interface.

## Methods and Materials

### Participants

Sixty-three infants between four and 5 months old, with and without CP, were recruited for this study involving the use of the SIPPC robot. Phase I recruited typically developing infants, and phase II recruited infants with or at high risk of developing CP. The inclusion criterion for infants in phase I was a motor development index (MDI) of at least 85 on the Bayley Scales of Infant and Toddler Development (3^rd^ ed., Bayley III) or at least a z-score of −1 standard deviation (sd) on the Test of Infant Motor Performance (TIMP) (Campbell et al., 2002; Kolobe et al., 2004). Inclusion criteria for infants in phase II were a TIMP z-score of −1 sd or lower, and MDI of 70 or lower, a confirmed diagnosis of CP, or MRI results indicating brain injury, prior to age two (Campbell et al., 2002; Kolobe et al., 2004). Upon entry into the study, parents of eligible infants consented their children and completed the Family Interview Form (FIF). The FIF was designed to collect demographic information and it included parents’ level of education, age, occupation, marital status, and household structure. Infant demographics included medical, health, birth, and developmental histories. The family demographic information collected from the FIF was not used in the data analyses as it did not directly measure the infants’ cognitive abilities or their motor learning and skill acquisition. The original study was reviewed by the University of Oklahoma IRB #5120 and the data analysis used de-identified data.

### Outcome Measures

The Bayley Scales of Infant and Toddler Development (3rd ed.) (Bayley, 2006), and the Movement Observation Coding Scheme (MOCS) (Rule, 2010) were the major outcome measures used in this study. The MOCS consists of 42 items and four subscales (Subscale 1: Posture and support, Subscale 2: Exploratory selection and progression, Subscale 3: Mastery of Propulsion, and Subscale 4: Socio-emotional responses). The MOCS measured the motor learning ability of the infants.

### Robotic Intervention Protocol

Infants from both phases completed the same protocol while using the SIPPC robot, involving two training sessions per week, up to 16 weeks, or until the crawling milestone was reached, where the crawling milestone was defined as the ability of the infant to crawl without assistance from the SIPPC robot or any other assistive device. Intervention began when infants reached 5–7 months of age, and occurred at either the infant’s home or the Human Development Laboratory at the University of Oklahoma Health Sciences Center. Therapists fitted infants with a securely strapped jumpsuit equipped with Inertial Measurement Unit (IMU) sensors. The intervention protocol involved the following steps:

1. Familiarization with the SIPPC robot—Infants played with both familiar and novel toys while being acclimated to the SIPPC robot for the first 1–2 min.
2. Assisted movement of infant’s arms and legs—If the infant was unable to initiate crawling toward a toy, investigators or caregivers moved the infant’s arms and legs to allow the infant to understand how to move the robot.
3. Self-initiated mobility—Researchers and caregivers encouraged infants to move the SIPPC using toys as a reward, for up to 5 min. If the infant was unable to move, researchers repeated step 2.

If the infant was able to crawl without assistance at or before the 16 weeks, i.e., if the crawling milestone was achieved, the infant no longer needed assistance from the SIPPC robot, thus concluding their participation in the study. Research staff repeated the Bayley III either when the infant achieved independent crawling, or at the end of 16 weeks.

### Data Analysis Procedure

The two major variables of interest for this data analysis were *cognitive ability* as measured by the mental development index of the Bayley-III (MDI) and *motor function* as measured by the MOCS. Infants were categorized into three distinct groups based on their baseline MDI scores: “above average” cognitive ability (group 1), “below average” cognitive ability (group 2), and “average” cognitive ability (group 3). Research staff calculated tri-weekly average scores on each subscale of the MOCS tool and used these as the outcome/dependent variable for all analyses. We measured change over time using five measurement time points containing the mean tri-weekly MOCS. We used variables indicating the crawling status of the infant and the length of follow-up/duration of intervention to conduct a survival analysis. This analysis allowed us to identify the percentage of infants who successfully achieved independent crawling (time to crawl variable), or the end of a 16-week intervention, whichever came first.

### Statistical Analysis

We computed summary statistics for variables within each “cognitive ability” group as well as the entire sample. We utilized repeated measures analysis of variance (repeated measures ANOVA) with the MOCS scores (tri-weekly averages) as the dependent variable, and including both “group” and “time” as independent variables, using an “unstructured” covariance structure. We used survival analysis to determine the crawling status of infants at the end of the study, as wells as their “time to crawl.” Finally, survival plot analysis for the first three subscales of the MOCS (Subscale 1: Posture and support, Subscale 2: Exploratory selection and progression, and Subscale 3: Mastery of Propulsion) allowed us to determine significant differences between the three cognitive groups of infants. Statistical procedures were repeated for all subscales of the MOCS except subscale 4 (“socio-emotional responses”) which we excluded from all analyses as this subscale does not provide information about motor learning ability of infants. We plotted mean MOCS scores over the five time points (representing weeks 1–3, 4–6, 7–9, 10–12,13–15) for each of the three subscales of the MOCS. All analyses utilized SAS 9.4 (Carey, NJ) with alpha = 0.05.

## Results

Forty-nine infants completed 16-week videotaped intervention sessions, complete with movement coding. One of these infants did not complete the study and another had incomplete cognitive scores at baseline, resulting in 47 infants included in the final analyses. Table 1 contains mean age, birth weight, gestational age, and baseline Bayley III cognitive scores.

**TABLE 1:** Mean and standard deviation values for demographic and baseline cognitive scores for all infants (*n* = 47), infants in the above average (*n* = 11), below average (*n* = 10), and average (*n* = 26) cognition groups.

Demographics/Group	All participants	“Above average” cognitive group	“Below average” cognitive group	“Average” cognitive group
N	47	11	10	26
Age at baseline (months)	4.66 (0.60)	4.45 (0.69)	4.90 (0.57)	4.65 (0.56)
Weight (lb)	3.71 (2.27)	3.43 (1.25)	4.96 (3.28)	3.29 (1.93)
Gestational age (weeks)	31.31 (5.12)	31.87 (3.50)	32.34 (6.95)	30.69 (4.95)
Cognitive score (Bayley III)	99 (18.57)	119 (3.93)	70 (14.34)	102 (6.19)

Although the mean MOCS subscale scores at baseline appeared different for infants in the “below average” cognitive ability group, and contained a higher degree of variability, none of the baseline scores were significantly different in any of the three subscales (*p* = 0.0523, 0.6819, and 0.3003 for subscales one, two and three, respectively) (Table 2).

**TABLE 2:** MOCS subscale scores for the three cognitive groups at baseline: “above average” cognition group (*n* = 11), “average” cognition group (*n* = 26), and “below average” cognition group (*n* = 10).

Group (based on cognitive ability)	Mean	Median	Std dev	*N*	Min.	Max.	Lower 95% CL for mean	Upper 95% CL for mean	*p*-Value
Subscale 1: Posture and support									
“Above average” cognition	7.0	7.0	2.1	11	3.0	10.0	5.6	8.5	0.0523
“Average” cognition	7.0	7.0	2.0	22	3.7	10.0	6.1	7.9	
“Below average” cognition	5.3	5.5	1.6	10	2.3	8.0	4.1	6.4
**Subscale 2: Exploratory selection and progression**									
“Above average” cognition	17.2	17.3	1.9	11	13.5	20.3	15.9	18.4	0.6819
“Average” cognition	15.3	16.7	5.9	22	3.0	25.0	12.7	17.9	
“Below average” cognition	15.5	14.1	8.4	10	5.5	36.0	9.5	21.5	
**Subscale 3: Mastery of propulsion**									
“Above average” cognition	20.8	21.0	4.2	11	13.7	27.5	17.9	23.6	0.3003
“Average” cognition	17.2	17.8	9.9	22	-3.5	35.0	12.8	21.6	
“Below average” cognition	14.6	16.3	10.8	10	-5.5	28.7	6.9	22.3	

### Time Differences

**FIGURE 1:**
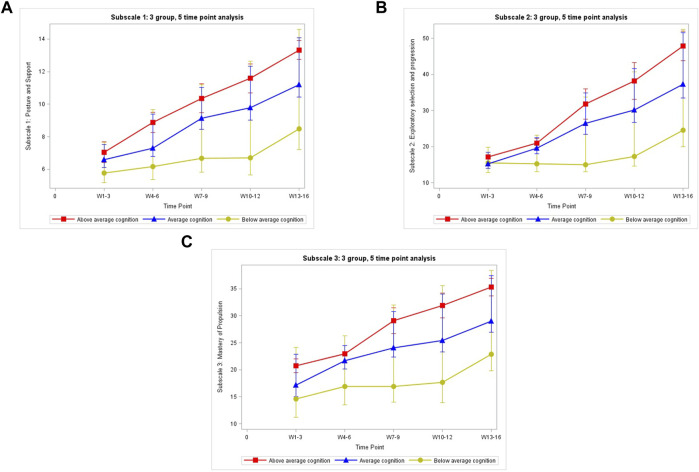
The mean MOCS scores for each group over the five time points for each subscale: **(A)** Subscale 1:3 group, 5 time point analysis. **(B)** Subscale 2:3 group, 5 time point analysis. **(C)** Subscale 3:3 group, 5 time point analysis.

The repeated measures ANOVA results demonstrate that *within all three* subscales, infants improved their scores at *each time point* (*p* < 0.0001), with the exception of time “2” in Subscale 1: Posture and Support (*p* = 0.1355). Plots of the mean MOCS scores for each group over the five time points for each subscale are displayed in Figures 1A–C (Table 3).

**TABLE 3:** Repeated measures ANOVA for each subscale (alpha = 0.05).

**Subscale 1: Posture and support**					
**Effect/Time/Group**	**Standard Error**	**t Value**	**Pr > |t|**	**Beta Estimates**	**95% CI for the Beta Estimates**
Intercept	0.4032	17.09	<0.0001*	6.8731	6.0624, 7.6838
Time 1 (weeks 1–3)—referent time					
Time 2 (weeks 4–6)	0.3498	1.52	0.1355	0.5320	−0.1730, 1.2369
Time 3 (weeks 7–9)	0.5074	4.50	<0.0001*	2.2840	1.2614, 3.3065
Time 4 (weeks 10–12)	0.6021	4.96	<0.0001*	2.9865	1.7730, 4.2000
Time 5 (weeks 13–160	0.6396	7.29	<0.0001*	4.6622	3.3732, 5.9512
“Average” cognition group—reference group					
“Above average” cognition group	0.7139	0.24	0.8103	0.1724	−1.2665, 1.6112
“Below average” cognition group	0.7379	−2.20	0.0331*	−1.6231	−3.1102, −0.1359
Significant interaction between time 2 and “above average” cognition group (group 1) (time*group)					
Time 2*group 1	0.6154	2.11	0.0401*	1.3014	0.0612, 2.5416
**Subscale 2: Exploratory selection and progression**					
**Effect/Time/Group**	**Standard Error**	**t Value**	**Pr > |t|**	**Beta Estimates**	**95% CI for the Beta Estimates**
Intercept	1.2068	12.33	<0.0001*	14.8770	12.4449, 17.3091
Time 1 (weeks 1–3)—reference time					
Time 2 (weeks 4–6)	1.0402	4.35	<0.0001*	4.5201	2.4238, 6.6164
Time 3 (weeks 7–9)	2.5160	4.43	<0.0001*	11.1520	6.0813, 16.2227
Time 4 (weeks 10–12)	2.9394	5.08	<0.0001*	14.9412	9.0172, 20.8652
Time 5 (weeks 13–160	3.0033	7.44	<0.0001*	22.3314	16.2785, 28.3842
“Average” cognition group—reference group					
“Above average” cognition group	2.1506	1.06	0.2960	2.2745	−2.0598, 6.6088
“Below average” cognition group	2.2231	0.29	0.7749	0.6397	−3.8406, 5.1200
Significant interaction between time (time 2, 3, 4, and 5) and “below average” cognition group (group 2) (time*group)					
Time 2*group 2	1.9063	−2.49	0.0165*	−4.7534	−8.5954, −0.9114
Time 3*group 2	4.7039	−2.47	0.0175*	−11.6187	−21.0987, −2.1387
Time 4*group 2	5.5326	−2.38	0.0215*	−13.1912	−24.3414, −2.0410
Time 5*group 2	5.6720	−2.34	0.0241*	−13.2480	−24.6792, −1.8169
**Subscale 3: Mastery of propulsion**					
**Effect/Time/Group**	**Standard Error**	**t Value**	**Pr > |t|**	**Beta Estimates**	**95% CI for the Beta Estimates**
Intercept	1.7146	10.30	<0.0001*	17.6558	14.2002, 21.1114
Time 1 (weeks 1–3)—reference time					
Time 2 (weeks 4–6)	0.7643	4.65	<0.0001*	3.5529	2.0125, 5.0932
Time 3 (weeks 7–9)	1.2088	5.29	<0.0001*	6.3999	3.9637, 8.8362
Time 4 (weeks 10–12)	1.4358	5.61	<0.0001*	8.0592	5.1656, 10.9528
Time 5 (weeks 13–160	1.2790	9.38	<0.0001*	12.0018	9.4243, 14.5794
“Average” cognition group—reference group					
“Above average” cognition group	2.6821	1.11	0.2750	2.9649	−2.4405, 8.3704
“Below average” cognition group	2.7743	−1.98	0.0543**	−5.4856	−11.0768, 0.1056

*means statistically significant, i.e., *p* < 0.05; **means trending towards statistical significance.

### Group Differences

Examination of group differences within subscale 1, “posture and support,” reveal that only infants in the “below average” cognition group (*n*_2_ = 10, mean = 5.3) were different from infants in the reference group, i.e., the “average” cognition group (*n*_3_ = 26, mean = 7.0) with *p* = 0.0331. No differences between group means were noted in subscale 2, “exploratory selection and progression,” or in subscale 3, “mastery of propulsion.”

### Interaction Effects

In the analyses of subscale 1, the “posture and support” subscale of the MOCS, there was only one interaction effect between the “above average” cognition group (*n*_1_ = 11) at time “2” (*p* = 0.04) when compared to the “average” cognition group (*n*_3_ = 26, reference group). In the analyses of Subscale 2, “exploratory selection and progression” subscale of the MOCS, there was statistically significant interaction between the “below average” cognition group and all time points i.e., times “2”, “3”, “4”, and “5” when compared to the “average” cognition group (*n*_3_ = 26, reference group) and baseline time point (time “1”). Finally, in the analyses for subscale 3, “mastery of propulsion” subscale of the MOCS, there was no statistically significant interaction between group and time. We excluded all insignificant interaction terms from the final model for each subscale (Table 3).

**FIGURE 2:**
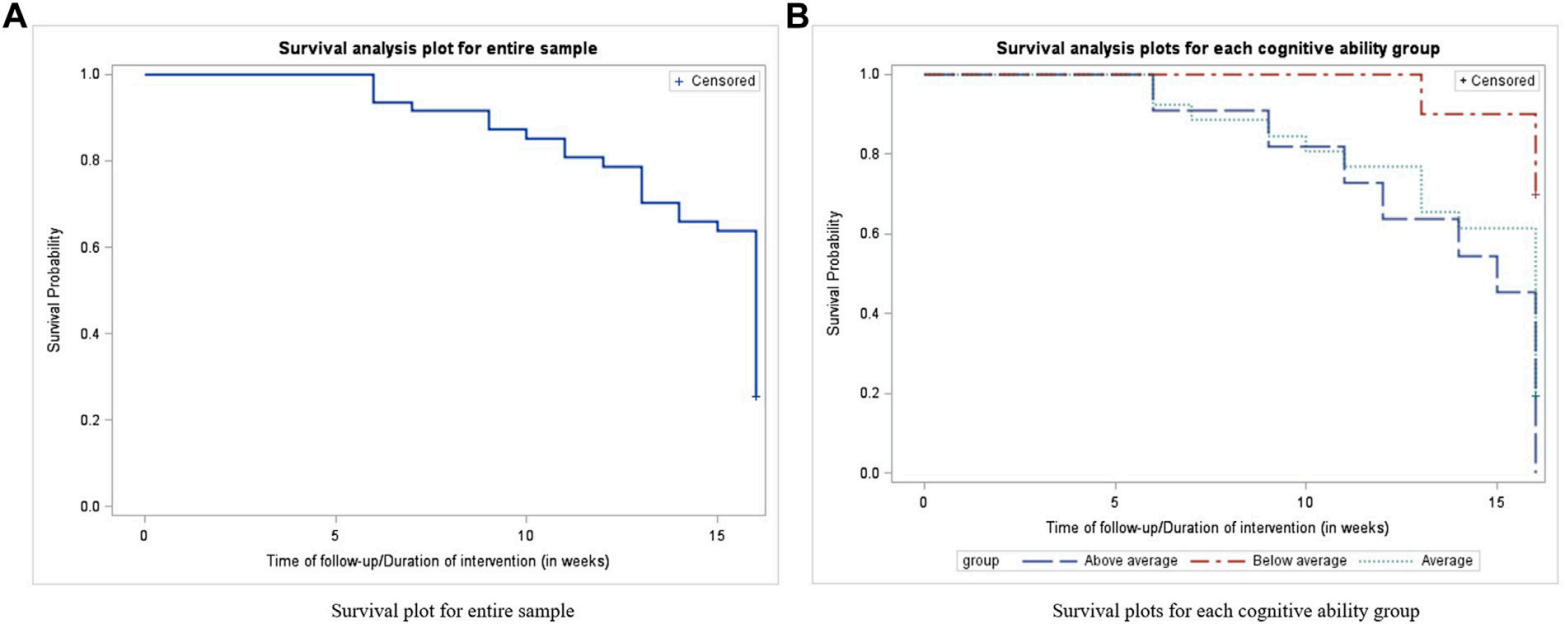
Successfully achieved the crawling milestone within or at the end of the 16-week intervention using the SIPPC robot based on survival analysis: **(A)** Survival plot for entire sample. **(B)** Survival plots for each cognitive ability group.

Survival analysis revealed 35/47 (74.5%) of the infant participants successfully achieved the crawling milestone by the end of the 16-week intervention. Comparing groups, 100% of the infants in the “above average” cognition group, 30% of the infants in the “below average” cognition group, and 80.8% infants in the of the “average” cognition group, successfully achieved the crawling milestone within or at the end of the 16-week intervention using the SIPPC robot (Table 4; Figure 2). Infants in both, the “above average” and “below average” cognition groups demonstrated significant differences (*p* < 0.0001) in their ability to achieve the crawling milestone when compared to the infants in the “average” cognition group within the 16-week intervention period. The odds of infants in the “above average” cognition group achieving crawling within the 16-week SIPPC intervention are 1.48 (1.23, 1.77) times higher than infants in the average group. The odds of infants in the “below average” cognition group achieving crawling the 16-week SIPPC intervention are 0.28 (0.20, 0.37) times lower than the infants in the average group.

**TABLE 4:** Survival analysis results using time to crawl, including the number of infants in each group who achieved the crawling milestone (*n* = 47).

Group	*N*	Crawled	Number who failed to crawl	Percent who failed to crawl	Likelihood of achieving crawling milestone within 16 weeks
“Above average” cognition group	11	11	0	0.00	1.48 (1.23, 1.77)
“Below average” cognition group	10	3	7	70.00	0.28 (0.20, 0.37)
“Average” cognition group	26	21	5	19.23	Reference group
Total	47	35	12	25.53	NA

## Discussion

The purpose of this study was to examine and evaluate the impact of cognitive ability of infants with and without CP on their ability to learn to use SIPPC robot. While baseline motor performance on the SIPPC (MOCS scores) between the three cognitive groups was not different, we found the rate of change of infants with lower cognitive ability differed from those with “average” or “above average” cognitive ability. Additionally, variability in the MOCS scores was higher in infants with lower cognitive ability when compared to infants with “average” or “above average” cognitive ability in all three subscales of the MOCS. These findings suggest that infants with lower cognitive ability took longer to master the motor skills involved in mobilizing the SIPPC robot than those with “average” or “above average” cognitive ability. Because the MOCS measures key movement strategies required to successfully learn to use the SIPPC robot, findings of this study corroborate results obtained from previous studies on the use of the SIPPC robot as an intervention for infants with or at risk of CP (Kolobe et al., 2015; Kolobe and Fagg, 2019).

In all three subscales of the MOCS, infants learned to use the SIPPC robot despite differences in cognitive status. Subscale one of the MOCS, “posture and support,” measures how long an infant is able to maintain an upright head position. In this study, infants with “below average” cognitive ability were unable to maintain an upright head position for as long a duration as those with “average” cognitive ability. Because infants were challenged to maintain an upright head position in response to the presentation of toys that held their interest, this finding could suggest higher cognitive ability was linked to greater interest in the toys used. This in turn facilitated enhanced motor learning effort and ability. The positive interaction term also indicates infants with “above average” cognition learn to keep their heads up faster than those in the “average” range. This may also reflect early enhanced interest in interacting with toys.

MOCS subscale 2 (exploratory selection and progression) measures the frequency of arm and leg use, driven by motivation to get to the toys used during the SIPPC robot intervention sessions. Higher frequency of arm and leg movements indicate the infant”s efforts to move the SIPPC robot towards the toy, thus is a good indicator of movement coordination and problem-solving strategies. Our results indicate infants with “below average” cognitive ability did not only show fewer movement related problem-solving strategies with their arms and legs than infants with “average” cognitive ability, their learning curve was also slower. The lack of change in subscale two MOCS scores of infants in the below average cognitive group, especially over the first 9 weeks (3 time points) of intervention (Figure 1B) is consistent with other findings that support the association between delayed motor function and cognitive ability (Monteiro et al., 2010; Robert et al., 2013; Kolobe and Fagg, 2019). The gradual increase in MOCS scores during the last 3 weeks of training demonstrate children with below average cognitive ability may benefit from prolonged practice.

The MOCS subscale 3 (mastery of propulsion) measures the total number of trials required for an infant to develop goal-oriented movements of the SIPPC robot before reaching the toy during intervention. This subscale also captures a degree of precision, error rate, and lack of effort. MOCS scores for infants with “below average” cognition changed at a slower rate than those with “average” or “above average” cognition. The slow rate of change (Figure 1C) indicates the presence of a delayed response time and a relatively higher and prolonged trial-and-error rate, shown to be associated with cognitive ability (Adolph, 2008; Middleton and Schwartz, 2012).

The survival analysis results indicate a relationship between crawling and cognition. This finding is consistent with existing literature on crawling (Anderson et al., 2001; Adolph, 2008; Anderson et al., 2013) Although not the primary focus of this study, all infants with above average cognition attained independent crawling before or by the end of the SIPPC robot intervention compared to 30% in the group with below average cognition. The lower performance on the key movement requirement for moving the SIPPC sheds light on this relationship that has not been previously reported. It appears that ability to maintain head position to engage toys visually and the problem-solving needed to coordinate arm and leg movement to reach toys, which require cognitive ability, also play a major role in the SIPPC robot design and development.

Although the main variables of interest in this study were the cognitive and motor learning ability of infants, one major confounder is the motor ability level of the infant, which might also impact the learning outcomes using the SIPPC robot. The primary purpose of this study was to evaluate whether the SIPPC robot was designed to cater to infants with varying levels of cognitive ability. The Bayley III motor ability scores show that, among infants with “below average” cognitive ability, those with higher levels of motor ability still successfully achieved the crawling milestone. This finding is an indication that the SIPPC robot is well-matched to cognitive ability. We hypothesize failing to achieve the crawling milestone within 16 weeks of using the SIPPC robot can be explained by a poor motor ability level of the infants at baseline. The findings from this study will be instrumental in further modifying and refining the design of the SIPPC robot. Specifically, the SIPPC could better match the motor ability level of the infants by providing additional physical assistance. Future versions of the SIPPC robot can also be made more sensitive to smaller movements to help infants with lower muscle strength move the robot more easily.

## Conclusion

The findings in this study reveal the key movement strategies required to move the SIPPC robot, assessed by the MOCS, vary depending on the infants’ cognition. While the results indicate a strong correlation between the infants’ cognitive ability and motor learning ability (MOCS scores), it does not support a causal link between the two variables (infant cognition and motor learning ability). The findings also suggest that the design and development of the SIPPC robot allows successful use by infants with varying levels of cognitive ability, although infants with lower cognitive ability require a longer duration of training to successfully improve their motor skills. This study did not examine the contribution of gross motor development status to learning to use the SIPPC robot and to cognitive ability. Future studies looking at ability to learn to use the device among infants with varying levels of motor ability and cognitive are warranted.

## Data Availability

The data analyzed in this study is subject to the following licenses/restrictions: The dataset can be shared with the approval from OUHSC IRB and the PI of the study (TK who is the senior author for this article). Requests to access these datasets should be directed to TK, Hlapang-Kolobe@ouhsc.edu.
